# The Science of Solid Lipid Nanoparticles: From Fundamentals to Applications

**DOI:** 10.7759/cureus.68807

**Published:** 2024-09-06

**Authors:** Navaneetha Krishnan M, Sangeetha S, Sree Ranjani P, Damodharan Narayanasamy

**Affiliations:** 1 Pharmaceutics, SRM College of Pharmacy, SRM Institute of Science and Technology, Chengalpattu, IND

**Keywords:** applications of slns, drug delivery, drug delivery system, nanocarriers, nanostructured lipid carriers, solid lipid nanoparticles (slns)

## Abstract

Solid lipid nanoparticles (SLNs) play a crucial role in drug delivery, offering benefits such as enhanced bioavailability, targeted distribution, and reduced toxicity. This article provides a comprehensive overview of SLN formulation, development, and advancement in pharmaceutical research, examining their characteristics, classifications, and significance. The review also delves into the real-world applicability of various SLN formulations across different routes of administration, discussing their advantages, disadvantages, and challenges of scalability, along with strategies for efficient implementation. Furthermore, it explores the diverse applications of SLNs through various delivery methods, addressing the obstacles and potential solutions. By highlighting the critical role of SLNs in improving treatment outcomes, this review underscores their importance in modern drug delivery systems.

## Introduction and background

Solid lipid nanoparticles (SLNs), once called lipospheres, are a promising type of pharmaceutical nanocarrier designed for the controlled release of medications. They measure under 50 nm to 500 nm [1]. SLNs are created by incorporating cationic lipids into liposomes, which then use electrostatic interactions to enclose negatively charged oligonucleotides. By fusing several of the beneficial aspects of polymeric nanoparticles, SLNs effectively address several limitations associated with conventional drug delivery systems (DDSs), such as suboptimal bioavailability, the need for controlled release, and reduced dosing requirements compared to standard formulations. The main components of SLNs are solid lipids, mostly physiological lipids, dispersed in an aqueous solution containing a surface stabilizer. As submicron colloidal carriers, SLNs are believed to offer the combined benefits of polymeric nanoparticle systems, fat emulsions, and liposomes while avoiding some of their disadvantages. To achieve high bioavailability, any delivery system needs to be hydrophilic (capable of dissolving in digestive fluids) and lipophilic (able to traverse lipid-based bio-membranes). SLNs are one of those few delivery systems that are biocompatible, highly stable, easily scalable, versatile, and can be used as a targeted delivery system [2]. SLNs were first identified by Müller and Lucks in the early 1990s, with their production method detailed in a patent application. However, the term SLN was coined a few years later by the same researchers when they described a technique for incorporating magnetite into nanoparticles. SLNs remain stable at both room and body temperatures. The lipid phase of SLNs is typically made from steroids, di- or tri-glycerides, maintaining a solid state at room and body temperatures. The surfactant concentration ranges from 0.5% to 5% (w/v) and falls under the generally recognized as safe (GRAS) category I. Using a combination of surfactants can enhance the stability of the SLN. The structure of SLNs is influenced by various factors, including the formulation components, the solubility of the compounds, such as the drug, and the production method. SLNs have emerged as a promising nanocarrier system. Compared to conventional colloidal carriers, SLNs exhibit lower toxicity, increased surface area, biocompatibility, extended drug release, enhanced cellular absorption, and improved drug solubility and bioavailability [3]. This narrative review provides a comprehensive analysis of the diverse formulation techniques employed in the preparation of SLNs. It highlights the versatility of SLNs as a DDS for both synthetic pharmaceuticals and phytopharmaceuticals. The review also explores the various drug administration routes applicable to SLNs and concludes by addressing the challenges associated with their use, along with strategic approaches for overcoming these obstacles.

## Review

Methodology

The literature selection and analysis for this review followed a systematic methodology. The search was conducted across multiple academic databases, including PubMed, Scopus, Web of Science, and Google Scholar, chosen for their extensive coverage of biomedical, pharmaceutical, and nanotechnology fields. To ensure a comprehensive collection of relevant studies, keywords such as "solid lipid nanoparticles," "SLNs," "drug delivery systems," "formulation techniques," "phytopharmaceuticals," "synthetic pharmaceuticals," "administration routes," "bioavailability," "nanoparticles characterization," and "herbal SLNs" were used. The search was refined with Boolean operators (AND, OR, NOT) to narrow down the results. Articles were selected based on specific criteria: they had to be peer-reviewed, published in English, and directly relevant to the formulation, characterization, and application of SLNs. Priority was given to recent publications, primarily from the past two decades, to ensure the inclusion of the most up-to-date information. Both original research articles and review papers were included to provide a well-rounded perspective. The selected literature was then categorized by key focus areas, including formulation techniques, DDSs, administration routes, and challenges in SLN application. A thematic analysis was conducted to identify trends, challenges, and innovations in each category.

Types

The drug's release from the formulation is affected by the type of matrix and the placement of the drug.

Drug-Enriched Shell Matrix

The drug-rich shell matrix emerges under conditions where the active pharmaceutical ingredient (API) concentration in the melted lipid is relatively low. During the cooling phase post-hot high-pressure homogenization (HPH), the lipid phase solidifies first, leading to a progressive concentration of the API in the remaining lipid melt. This process results in the formation of a core that is largely devoid of API, around which a shell containing both API and lipid crystallizes as the API reaches its saturation solubility. This model is particularly advantageous in scenarios where a burst release of the API is desired, such as in immediate therapeutic interventions. Additionally, the occlusive properties of the lipid core can be leveraged to enhance the skin penetration of topically applied formulations.

Homogenous Matrix

A homogeneous matrix involves the API being uniformly dispersed at a molecular level throughout the lipid matrix or present in amorphous aggregates. This configuration is typically achieved by employing precise ratios of API to lipid, followed by HPH conducted at temperatures exceeding the lipid’s melting point, or alternatively, through the cold HPH technique. The homogeneity of the lipid matrix in SLN Type I plays a crucial role in enabling controlled and sustained drug release, making it particularly valuable in applications requiring prolonged therapeutic effects and enhanced bioavailability.

Drug-Enriched Core Matrix

**Figure 1:**
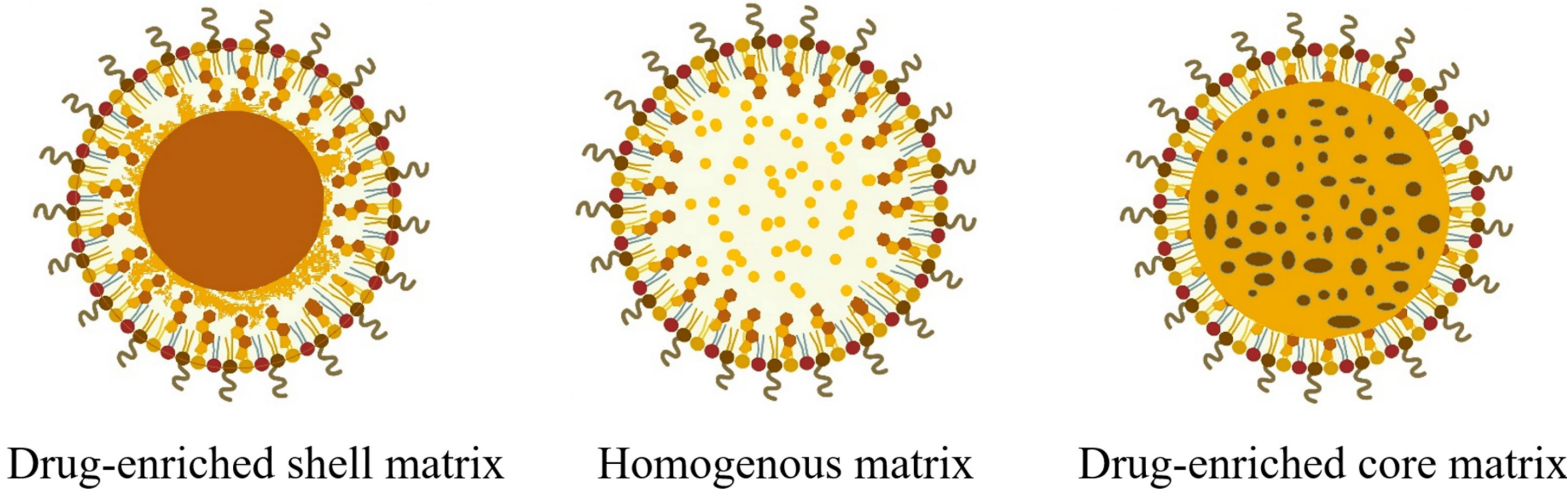
Solid lipid nanoparticles Image credit: Dr. Navaneetha Krishnan M

The drug-rich core matrix is established when the drug is dissolved near its saturation solubility in the lipid melt, leading to super-saturation upon cooling. Super-saturation is a state where the concentration of the drug exceeds its solubility limit in the lipid, making the solution unstable. This super-saturation triggers the drug to recrystallize, a process where the dissolved drug molecules revert to their solid crystalline form prior to the lipid, resulting in a core that is densely packed with the drug. Subsequent cooling causes the lipid to recrystallize around this drug-rich core, forming a protective barrier. This model is highly effective for achieving prolonged drug release, as the lipid barrier can slow down the diffusion of the drug, thereby extending its therapeutic window. Moreover, the controlled release properties of this model can be fine-tuned by modifying the composition and crystallization behavior of the lipid matrix, providing a versatile platform for a wide range of pharmaceutical applications [4,5]. Figure 1 provides the most relevant and accurate graphical depiction of the three types of matrix models in SLNs.

Advantages

SLNs serve as effective carriers for various active substances, offering numerous advantages in DDSs. These carriers can be administered via multiple routes. The physiological nature of SLN compounds allows for targeted delivery by overcoming biological barriers, thereby reducing the risk of both acute and chronic toxicity. SLNs offer numerous benefits for drug delivery. They can control and sustain drug release, enhancing therapeutic effects and reducing the need for frequent drug administration. SLNs also improve the bioavailability of lipophilic drugs, making them more effective. As they are made from biocompatible and biodegradable lipids, SLNs are generally safe and minimize side effects. They protect drugs from degrading due to chemical and physical factors, ensuring stability. Furthermore, their small size enables targeted drug delivery, which can reduce harm to healthy tissues and boost treatment efficiency [6].

Disadvantages

However, SLNs also have their downsides. The production process is complex and expensive, requiring specialized equipment and materials. Stability issues can arise, such as drugs being expelled during storage or changes in the lipid structure that affect reliability. Encapsulating some drugs can be inefficient, which is particularly pronounced when dealing with drugs that have large molecular sizes, high melting points, or are sensitive to thermal and mechanical stress. Furthermore, encapsulation efficiency may be compromised under conditions such as suboptimal pH, temperature variations, or the presence of impurities in the raw materials, all of which can destabilize the nanoparticle structure and lead to premature drug release. Scaling up to industrial production poses additional challenges. Additionally, the lipids and surfactants used in SLNs might be toxic, requiring careful testing. While SLNs are promising, addressing these challenges through ongoing research is essential for their practical use.

Methods for preparing SLNs

Homogenization Method for SLNs

**Figure 2:**
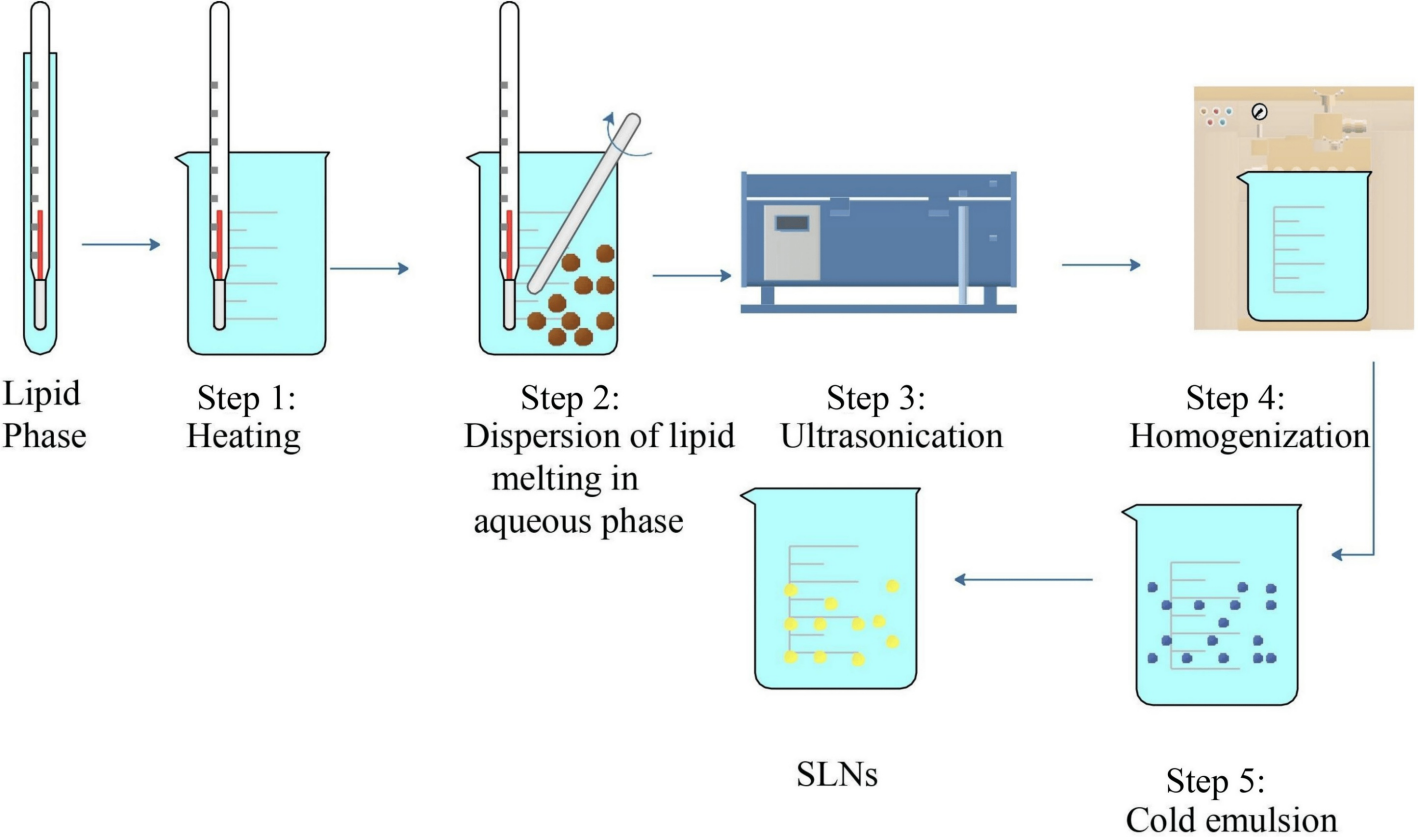
Hot homogenization Image credit: Dr. Navaneetha Krishnan M SLNs: Solid lipid nanoparticles

**Figure 3:**
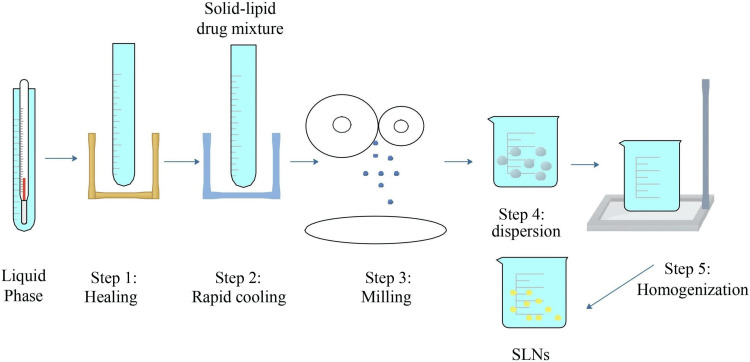
Cold homogenization Image credit: Dr. Navaneetha Krishnan M SLNs: Solid lipid nanoparticles

Homogeneous SLNs are a specialized type of nanoparticle system used to deliver drugs. They are made from solid lipids, providing a stable structure to encase APIs. The term "homogeneous" here means that each batch of SLNs has a consistent particle size and composition. This uniformity is vital for ensuring drugs are released predictably and effectively. Homogeneous SLNs are favored because they offer precise control over how drugs are released and how stable they remain, which is crucial for their therapeutic benefits. Homogeneous SLNs are the most widely used method due to their easy scalability and uniformity [7]. The discussion of homogeneous SLNs in various fields is shown in Figures 2-3.

Single and Double Emulsions Solvent Evaporation Method for SLNs

The single emulsion solvent evaporation step begins by dissolving the lipid in an organic solvent. If there is a drug to be encapsulated, it is also dissolved in this organic phase. This lipid-drug hybrid is then emulsified into an aqueous phase with a surfactant. Usually, high-speed homogenization, followed by ultrasonication, is used to create oil-in-water (O/W) emulsions. The next step involves evaporating the organic solvent under reduced pressure while stirring the emulsion. As the solvent disappears, the lipid precipitates and forms SLNs. These nanoparticles are then collected by centrifugation or filtration and washed to remove any excess surfactant and non-encapsulated drug(s) [8].

The drug is first dissolved in an aqueous solution using double-distilled water. After that, this solution is injected dropwise through a needle at one end of a glass syringe barrel packed with lipids dissolved in dichloromethane, until all the solution is used up, leading to the formation of a water-in-oil (W/O) emulsion. This emulsion is subsequently added drop by drop into a larger volume of an aqueous phase containing surfactants [9]. 

Solvent Injection Method for SLNs

**Figure 4:**
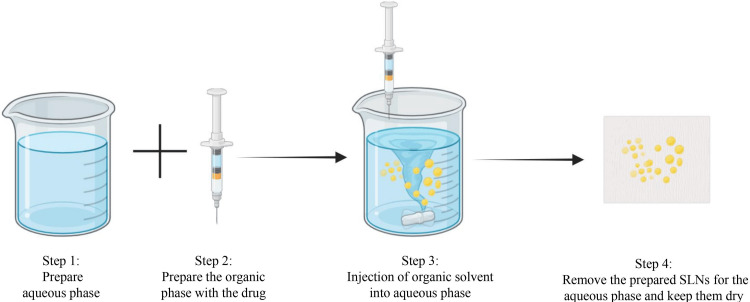
Solvent injection method Image credit: Navaneetha Krishnan M SLNs: Solid lipid nanoparticles

An efficient method often used in the preparation of SLNs involves injecting a solvent. Here, the lipids are dissolved in an organic solvent that is soluble in water, such as ethanol or acetone. This solution is then quickly injected into a stirred aqueous phase containing a surfactant or stabilizer. With the rapid dispersion and intermixing of the organic solvent with the aqueous phase, the lipid diffuses out, leading to its precipitation and the formation of nanoparticles. The rapid diffusion of the solvent makes these particles appear almost instantaneously. In subsequent stages, the organic solvent must be evaporated away by sublimation at reduced pressure or by dialysis to ensure that no traces of solvent remain in the final product. This method of producing SLNs possesses several merits, including simplicity, easy operation, and mild processing conditions, thus making it appropriate for temperature-sensitive drugs. Additionally, it can be easily extended for large-scale production purposes, which is advantageous. Nevertheless, for safety reasons, all traces of solvents must be carefully removed, while their physical solubility in organic compounds may limit their applicability when used as shell materials during polymerization procedures. Despite these hardships, the solvent injection method still represents an effective and economical way to produce SLNs [10]. Figure 4 represents the formulation methodology of preparing SLNs using the solvent injection method.

Micro/Nano Emulsions-Based SLNs

**Figure 5:**
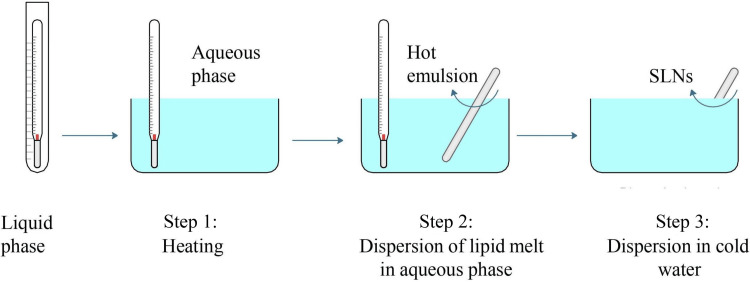
Micro emulsion Image credit: Navaneetha Krishnan M SLNs: Solid lipid nanoparticles

The single emulsion solvent evaporation process begins with micro- and nano-emulsions of SLNs, which are an advanced topic in DDS, combining the advantages of emulsions with the distinct features of SLNs. They have various advantages, including increasing the solubility of poorly water-soluble medicines, improving drug stability, and providing controlled release features. The particles' tiny size enables greater penetration and absorption in biological systems. However, the formulation and stability of these emulsions can be difficult, necessitating precise control over factors such as particle size, zeta potential, and surfactant concentrations. Furthermore, the biocompatibility and safety of these formulations must be rigorously tested prior to clinical application to ensure their efficacy and safety in human use [11]. The emulsion-based formulation of SLNs is discussed in Figure 5.

Solvent Diffusion-Based SLNs

The ease and efficacy of the solvent diffusion approach make it a popular technique for creating SLNs. A lipid is first dissolved in an organic solvent that is water-soluble, such as ethanol, acetone, or methanol. The solvent of choice is determined by the solubility of the lipid and the desired characteristics of the final nanoparticles. After that, while continuously stirring, the organic phase is gradually added to an aqueous solution containing a surfactant. Through its ability to lower surface tension and prevent aggregation, the surfactant helps to stabilize the nanoparticles. The lipid separates from the solution to produce SLNs when the organic solvent diffuses into the aqueous phase. The production of tiny, evenly-sized nanoparticles is the outcome of the solvent diffusing quickly [12].

Double Emulsion Solvent Displacement-Based SLNs

An improved method for efficiently encapsulating hydrophilic drugs is the creation of SLNs using the double emulsion solvent displacement process. Using this technique, a medication contained in an aqueous solution is dispersed in a lipid phase containing a water-immiscible organic solvent, to create a W/O primary emulsion. The next step involves adding this primary emulsion to an external aqueous phase that contains a surfactant, creating a double emulsion (W/O/W). As the organic solvent from the internal phase diffuses into the external aqueous phase, the lipid precipitates and solidifies as drug-encapsulating lipid nanoparticles. To create a stable dispersion of SLNs, the organic solvent must be eliminated in the final stage, which is usually accomplished by dialysis under low pressure [13].

Applications

In the review article by Ganesan et al. (2017), several research studies were referenced, highlighting various applications of SLNs with different combinations of lipids and surfactants to produce effective formulations. SLNs have demonstrated wide-ranging potential, and ongoing research continues to explore both synthetic and herbal drug formulations. The application of SLNs is extensive, and research continues to uncover new potential uses, showcasing their versatility and therapeutic efficacy. For instance, formulation of α-trans retinoic acid into SLNs using Compritol 888 ATO, Soy Lecithin/Pluronic F68, or Soy Lecithin/Tween 80 through HPH. This formulation significantly enhanced gastrointestinal absorption and oral bioavailability, which is crucial for the treatment of acne and certain cancers. Similarly, developing SLNs for α-asarone using glyceryl caprate and polyethylene glycol 660 hydroxystearate via ultrasonic homogenization. The study demonstrated improved oral bioavailability, tissue uptake, and distribution, which are vital for its neuroprotective effects. In the realm of muscle relaxants, prepared using stearic acid, Epikuron 200, propionic acid, butyric acid, and sodium taurocholate to create SLNs for baclofen using a multiple (W/O/W) warm microemulsion method. This formulation was found to enhance baclofen's bioavailability, making it more effective in treating spasticity. Similarly, SLNs prepared for buspirone HCl with acetyl alcohol/spermaceti and either Tween 20 or poloxamer using emulsification-evaporation followed by ultrasonication. This method led to a significant improvement in buspirone's oral bioavailability, optimizing its anxiolytic effects. Calcitonin, an essential drug for treating osteoporosis, was formulated into SLNs using trimyristin through the W/O/W emulsion technique. Their results indicated a marked improvement in the efficiency of oral delivery of this protein, which is critical for patient compliance. In the field of immunosuppressants, cyclosporin A was incorporated into SLNs using glyceryl monostearate and glyceryl palmitostearate via melt-homogenization with an HPH. This formulation achieved low variation in bioavailability and prevented the high initial plasma concentrations typically associated with this drug, which is essential in organ transplantation scenarios. Furthermore, Jain et al. (2010) utilized glyceryl caprate in a solvent emulsification-diffusion method to formulate doxorubicin SLNs, which enhanced apoptotic death in cancer cells, showcasing its potential in chemotherapy [10]. Gonadotropin-releasing hormone, used in reproductive health therapies, was formulated into SLNs using monostearin and monoglyceride through a novel solvent diffusion method, resulting in prolonged drug release. Insulin, a critical therapeutic for diabetes, was incorporated into SLNs using glyceryl monostearate, glyceryl palmitostearate, glyceryl tripalmitate, and glyceryl behenate via a double emulsion method, which showed promise for oral delivery. Finally, SLNs were prepared for N3-O-toluylfluorouracil, an anticancer agent, using ATO888, soya lecithin, and TFU with a film dispersion-ultrasonication technique, resulted in enhanced GI absorption and improved oral bioavailability, thereby optimizing the drug's anticancer efficacy [5].

The integration of herbal drugs into SLNs has shown significant potential across a range of therapeutic applications. Various herbal extracts, when combined with specific lipid polymers, have demonstrated enhanced bioactivity through different preparation methods. For instance, *Hibiscus rosa-sinensis*, formulated with glyceryl monostearate via the emulsification-hot melt homogenization method, has shown promise in treating depression. Similarly, pomegranate extract, when encapsulated in stearic acid and Precirol ATO 5 using the hot homogenization and ultra-sonication method, exhibits potent antioxidant activity. The anti-inflammatory properties of triptolide have been effectively harnessed by incorporating it into tristearin glyceride and stearic acid through probe sonication. *Calendula officinalis*, using a warm microemulsion technique, has been noted for its re-epithelialization capabilities, while the combination of Frankincense and Myrrh in glyceryl dibehenate via HPH shows antitumor efficacy. Neem oil incorporated with cholesterol using a double emulsification method has been explored for its anti-*Toxoplasma* properties. Furthermore, *Annona muricata*, formulated with stearic acid through HPH followed by ultrasonication, has demonstrated anticancer activity. The respiratory tract infections are addressed by Yuxingcao using glyceryl behenate via high-shear homogenization. Lastly, andrographolide combined with stearic acid and glyceryl monostearate through a modified solvent injection technique also exhibits promising anticancer properties. These findings underscore the growing importance of SLNs in DDSs, as they offer significant improvements in the therapeutic outcomes of both synthetic and herbal drugs [2].

SLNs in various routes of administration

SLNs are among the few DDSs that offer high biocompatibility due to their lipid-based composition, which is similar to the natural lipids in the human body, thereby reducing the likelihood of an immune response. Their stability is another significant advantage, not just because of the use of surfactants, which help maintain the structural integrity of the nanoparticles by preventing aggregation, but also due to other factors such as the type of lipid used, the method of preparation, and storage conditions. Surfactants play a critical role in forming a protective layer around the lipid core, contributing to the overall stability and ensuring the SLNs remain physically and chemically stable over time, preventing drug leakage and maintaining their efficacy. In the review article by Akbari et al. (2022), SLNs have been investigated for various administration routes, each with its own set of benefits and challenges. In topical applications, SLNs enhance drug permeation by bypassing the stratum corneum and utilizing pathways like follicles or transcellular and paracellular routes. cryoprotectants were used during the lyophilization of topical amphotericin B SLNs, where stability was significantly improved. For oral delivery, SLNs help overcome low bioavailability due to hepatic first-pass metabolism and limited drug solubility. Modifications such as chitosan coating have been shown to increase oral absorption. In ocular delivery, SLNs can bypass barriers like the ocular blood barrier and improve drug retention and bioavailability within the eye. indomethacin-loaded SLNs, with surface modifications, enhanced ocular penetration. For parenteral administration, SLNs can be administered via various routes, including intravenous and subcutaneous, providing benefits like prolonged drug release and extended circulation time. In pulmonary delivery, SLNs target drug release within the lungs, improving bioavailability and patient compliance. Research demonstrated the potential of SLNs for pulmonary applications, with high encapsulation efficiency and sustained drug release. Finally, SLNs show promise in brain delivery by crossing the blood-brain barrier, enhancing drug uptake, and providing controlled release. However, challenges such as burst drug release and potential toxicity need to be addressed for each administration route [14].

Obstacles and strategies

SLNs encounter various challenges, including complications such as polymorphism, phase separation, and sterilization difficulties during manufacturing. Polymorphism, which involves the formation of different crystalline structures, can be controlled using temperature-regulated techniques like supercritical fluid (SCF) technology. However, there are no documented efforts to scale up SLN production utilizing SCF. To address phase separation, which can undermine the stability of SLNs, an optimized lyophilization process is necessary. Lyophilization facilitates the creation of solid particles that are more stable and effectively minimize microbial growth. Sterilization is another challenge, particularly because thermolabile compounds and lipids are vulnerable to damage from gamma irradiation and steam sterilization. This challenge can be alleviated by employing filtration methods, which are less harsh on delicate materials. Additionally, optimizing production parameters is crucial to increase yield, reduce waste, and lower costs, which can be further achieved through continuous manufacturing processes that enhance efficiency and throughput. Stabilizers or co-surfactants can be used to prevent aggregation and strengthen the SLN structure, while controlling environmental factors like temperature and pH during storage is vital to maintaining stability. Furthermore, improving the selection of lipids that are more compatible with the drug and refining production techniques can enhance encapsulation efficiency. Scaling SLN production from laboratory to industrial levels is challenging due to differences in equipment, process parameters, and maintaining batch consistency. Issues such as uniform particle size, consistent drug loading, and batch-to-batch reproducibility must be addressed by adopting scalable methods like HPH, which can transition smoothly from small-scale to large-scale manufacturing. Rigorous process validation and quality control are essential to ensure consistent SLN properties across different production scales. Although SLNs are generally biocompatible, certain formulations may still pose toxicity risks, particularly at higher concentrations or with prolonged exposure. This toxicity could result from interactions between SLNs and cells, leading to membrane disruption, inflammation, or other adverse effects. To mitigate these concerns, careful selection of lipids and surfactants with established safety profiles, and thorough preclinical testing are recommended to assess the biocompatibility and toxicity of the final formulation [15].

Evaluation of SLNs

Entrapment Efficiency

Entrapment efficiency is defined as the ratio of the total quantity of drug encapsulated in the SLNs relative to the total quantity of drug introduced during formulation. This efficiency represents the total amount of drug present in the SLNs. Several factors can influence the drug entrapment. SLNs are manufactured by using lipids, and every lipid has its compatibility and encapsulating capacity; therefore, the selection of the lipids plays a significant role during the formulation. Additionally, surfactants, drug properties, and method of preparation will have a compelling impact on the formulation and drug delivery [16].

EE(%) = (Total amount of drug added/Amount of drug entrapped​) × 100

Particle Size Analysis

Particle size analysis is an important characterization process used to evaluate whether the SLNs are distributed equally throughout the formulation. It is a critical parameter that influences the stability, bioavailability, and overall effectiveness of the system. Many techniques are used to evaluate the particle size, which include dynamic light scattering (DLS), laser diffraction, transmission electron microscopy, scanning electron microscopy, and atomic force microscopy [17].

Polydispersity Index (PDI)

The PDI is a major parameter used to analyze the uniformity of the particle size. DLS is used to assess the variation in scattered light intensity caused by the suspended particles moving. It is a cumulative analysis where the width of the particle is analyzed along with the mean size. The usual PDI values range between 0 and 1, and anything above 0.3 is considered non-uniform in size. PDI values are often associated with the stability of the nanoparticles [18].

Zeta Potential

Zeta potential is a measure of the electrical potential at the surface of the SLNs relative to the dispersed medium. It is determined using electrophoretic light scattering. This involves calculating the velocity of particles migrating towards their opposite charge. It is measured in millivolts, and values greater than +30 mV or less than -30 mV are considered stable. Neutral to negative millivolts are generally considered weak electrostatic repulsions, which are likely to result in particle aggregation, while extremely high values are typically referred to as better dispersed and stable formulations [19].

Chemical Stability

Chemical stability is an important factor that ensures the drug and the lipid matrix do not undergo any chemical degradation. Differential scanning calorimetry (DSC) is an evaluation procedure that measures the heat capacity and thermal stability of the samples versus the reference material. It determines the melting point of the lipid components and also detects crystallization events. DSC is a parameter that reports the thermal stability, drug-lipid interaction, and physical state of lipids [20].

Drug Release Profile

The drug release profile evaluates the rate at which the drug is released from the formulation into its surrounding medium. It is often represented as mg/hr. The importance of the drug release profile is to evaluate its consistency and bioavailability. The testing methods can be performed using both in-vivo and in-vitro methods [21].

Future perspectives

The future prospects of SLNs in drug delivery are highly promising, particularly as ongoing advancements in nanotechnology and pharmaceutical sciences continue to refine their design and application. However, significant challenges remain in the scalability of SLN production, including issues related to batch-to-batch consistency, cost-effective manufacturing, and the complexity of formulation processes. To effectively reduce the cost of these novel delivery systems, researchers are exploring simplified methods of preparing SLNs, such as using standard, scalable, and efficient development procedures that leverage readily available and cost-effective materials. Innovations in HPH, solvent emulsification, and microemulsion techniques are being optimized to enhance production efficiency while maintaining the quality and stability of the nanoparticles. Additionally, integrating continuous manufacturing processes and automation could further reduce costs and facilitate large-scale production. As these challenges are addressed, SLNs are expected to become more accessible and viable for widespread clinical use, ultimately contributing to the development of more effective, affordable, and patient-friendly therapies.

## Conclusions

SLNs have since evolved into a versatile and promising formulation technique due to their unique properties. SLNs are formulated using physiological lipids, surfactants, and water. They offer several advantages, such as controlled and sustained drug release, improved bioavailability, increased stability, and biocompatibility. These advantages make SLNs suitable for various routes of administration, including intravenous (IV), intramuscular (IM), subcutaneous (SC), oral, ocular, and dermal. Despite their potential, SLNs are not without challenges. The complexity and cost of production, stability issues, and encapsulation efficiency pose significant hurdles. The diverse preparation methods - from homogenization and solvent evaporation to micro/nanoemulsion and solvent diffusion methods - highlight the adaptability and applicability of SLNs in pharmaceutical sciences, as well as the obstacles and strategies involved. As ongoing research continues to address these challenges, SLNs are poised to play an increasingly critical role in delivering therapeutic agents more effectively and safely, thereby enhancing patient care and treatment outcomes across various medical fields.
